# Drug utilization patterns before and during COVID-19 pandemic in Manitoba, Canada: A population-based study

**DOI:** 10.1371/journal.pone.0278072

**Published:** 2022-11-28

**Authors:** Laila Aboulatta, Payam Peymani, Christine Vaccaro, Christine Leong, Kaarina Kowalec, Joseph Delaney, Jamie Falk, Silvia Alessi-Severini, Basma Aloud, Sherif Eltonsy

**Affiliations:** 1 College of Pharmacy, Rady Faculty of Health Sciences, University of Manitoba, Winnipeg, Canada; 2 Department of Psychiatry, College of Medicine, Rady Faculty of Health Sciences, University of Manitoba, Winnipeg, Canada; 3 Department of Medical Epidemiology and Biostatistics, Karolinska Institutet, Stockholm, Sweden; 4 Department of Epidemiology, University of Washington, Seattle, WA, United States of America; 5 Manitoba Centre for Health Policy, Winnipeg, Canada; 6 The Children’s Hospital Research Institute of Manitoba, Winnipeg, Canada; St. Michael’s Hospital, CANADA

## Abstract

**Background:**

The COVID-19 pandemic has led the Canadian provincial governments to take unprecedented measures, including restrictions to healthcare services and pharmacists. Limited evidence exists on changes in prescription trends in Canada during the pandemic period.

**Objectives:**

To examine the trend of prescription medications’ utilization before and during COVID-19, among incident and prevalent users in the general population. We examined 18 major classes of medications.

**Methods:**

We used the administrative health databases from the province of Manitoba, Canada, to conduct a province-wide cross-sectional study. Incident and prevalent use was compared between two time periods; pre-COVID-19: July 2016-March 2020 and during COVID-19: April 2020-March 2021. Interrupted time series analysis using autoregressive models was used to quantify the change in level and slope in quarterly medication use among incident and prevalent users.

**Results:**

The quarterly study population ranged from 1,353,485 to 1,411,630 Manitobans. The most common comorbidities were asthma (26.67%), hypertension (20.64%), and diabetes (8.31%). On average, the pandemic restrictions resulted in a 45.55% and 12.17% relative decline in the aggregated utilization of all drugs among both incident and prevalent users, respectively. Subclass analysis showed a 46.83%, 23.05%, and 30.98% relative drop among incident users of antibiotics, cardiovascular drugs and opioids use, respectively. We observed a significant slope increase during COVID-19 among the quarterly cardiovascular, antidiabetics, alpha-1 blockers, and statins incident users compared to the pre-COVID-19 period. We noted a significant decrease in level among NSAIDs, opioids, and antibiotic prevalent users, however, no significant changes in slope were observed.

**Conclusion:**

Our findings show a significant impact of COVID-19 measures on prescription trends in the general population. The observed decline among several medication classes was temporary. Further research is needed to monitor prescription trends and better understand if those changes were associated with increased health services and worsened outcomes.

## Introduction

COVID-19 spread rapidly around the world, and the outbreak was declared a global pandemic by the World Health Organization on March 11, 2020 [1–3]. As of November 29, 2021, Canada has documented 1,790,142 cases of COVID-19, resulting in 29670 deaths [4]. In the Canadian province of Manitoba, the first COVID-19-positive case was identified on March 12, 2020 [5]. Lockdown measures were applied to reduce the peak virus activity in the community, beginning on March 16, 2020 [6]. However, the pandemic has led to changes in healthcare access within the province [7]. As for prescription medications access, the pandemic has interrupted supply chains on a global scale and affected millions of patients who need a continuous supply of medications to treat their chronic conditions [8]. Manitoban pharmacists were mandated to limit the quantity of drug dispensed to a one-month supply of medications to mitigate the risk of medication shortages [9]. However, this restriction was relaxed on May 11, 2020, allowing patients who are on long-term medications to receive a three-month supply, if the medication is not influenced by shortages [10].

Although it is likely that COVID-19 associated restrictions have impacted how individuals seek and receive medical care, their influence on resource utilization is still unclear. In particular, the impact of the COVID-19 pandemic on medication utilization patterns across age groups has not yet been fully elucidated. Studies showed conflicting results when evaluated the use of specific medication classes in certain patient groups after the start of the pandemic [8, 11–14]. In Ontario, few studies evaluated the impact of the pandemic on the utilization of direct acting antivirals [15, 16] and prescription trends among nursing home residents [11, 17].

Understanding the influence of COVID-19-related public health measures on medication utilization patterns is crucial, especially as means to support healthcare providers and policymakers to ensure applying best practices during future outbreaks and pandemics. The objective of this study was to examine trends in medication use among Manitobans before and after the implementation of COVID-19-related restrictions with a focus on commonly used and chronic diseases medications.

## Materials and methods

### Description of databases

We used data from the Manitoba Population Research Data Repository at the Manitoba Centre of Health Policy (MCHP). The Repository is a secure data-rich environment containing person-level health information on virtually the entire population of Manitoba. The validity and reliability of the MCHP Repository for epidemiological studies has been previously reported [18–20]. All records are de-identified and linkable at the individual and family levels using a scrambled health number. The Drug Programs Information Network (DPIN) database is a provincial pharmacy data source that allows all entered prescriptions in an ongoing cycle to be assessed for drug utilization trends. We used the following databases: the Manitoba Health Insurance Registry (for date of birth and sex); physician claims and hospital discharge abstracts (for contacts with the healthcare system and diagnoses using the International Classification of Diseases (ICD-9 or ICD-10 codes); postal codes from the Canada Census (to distinguish urban from rural regions), and census data (for income quintiles based on ranges of mean household income, and grouped into five categories with each quintile assigned to approximately 20% of the population).

### Study design and population

A longitudinal population-based cross-sectional study was used to examine utilization trends for selected medications before and during the COVID-19 pandemic. The study included all Manitoba residents (excluding medications prescribed during hospital stays) from July 1, 2016 to March 31, 2021. Subjects were compared between two time periods: Period 1 (pre-COVID-19: July 1, 2016, to March 31, 2020); and Period 2 (COVID-19: April 1, 2020 to March 31, 2021). A strict mitigation strategy was implemented in Manitoba from March 13, 2020 (first wave) followed by a subsequent ease of some restrictions between June and July 2020. More restrictions were applied during the second wave, with Manitoba meeting its peak lockdown stringency index of 81.8/100 in November 2020 [21, 22]. The Q2-2020 was chosen as the intervention point for COVID-19 period since the first case of COVID-19 was reported on March 12, 2020, and restriction measures started to impact clinical practice in Manitoba by the end of Q1-2020.

### Study medications and variables

We examined the quarterly medication prescriptions for the following medication classes: anti-diabetics, cardiovascular drugs, opioids, respiratory drugs, antibiotics, antivirals, corticosteroids, prescribed non-steroidal anti-inflammatory drugs (NSAIDs), chemotherapy drugs, immunostimulants, immunosuppressants, hydroxychloroquine, neuraminidase inhibitors, thyroid medications, proton pump inhibitors (PPI), statins, bisphosphonates, and alpha-1 adrenergic receptor blockers. We also assessed the quarterly medication prescriptions for “all drugs” defined as the prescribed drugs only from the medication classes captured in aggregate and “any drugs” which is defined as any prescribed drug from DPIN data during the study period. All medications were retrieved from the MCHP Repository based on Anatomical Therapeutic Chemical (ATC) codes and categorized by the therapeutic classes (**S1 Table**). Patients were considered to have been prescribed any of those medications if they had one or more prescriptions filled between July 1, 2016, to March 31, 2021. Quarterly dispensing rates for these medications were reported using data provided by DPIN for Period 1 and 2. Patient variables included sex, age (< 65 and ≥ 65 years old), household income, residence (urban and rural), and comorbid diseases including asthma, congestive heart failure, coronary artery diseases, diabetes, and hypertension.

### Statistical analysis

Incidence and prevalence of use were calculated for each medication class and overall. Incident users were defined as individuals who had not been dispensed the examined medication of interest in the previous 4 quarters to their first dispensation; whereas prevalent users were defined as individuals who used the medication within each quarter during the study period. Manitoba residents with at least one day of Manitoba Health and Seniors Care (MHSC) coverage during the quarter was the denominator. Patient demographics (age, sex, area of residence, and income quintile) and comorbid diseases (MCHP validated definitions of asthma [23], diabetes [24], coronary artery diseases [25], congestive heart failure [26], and hypertension [27]) were described before and during the pandemic.

The quarterly incidence and prevalence of all medication use were calculated. We conducted interrupted times series analysis using non-linear regression with autoregressive terms, a powerful quasi-experimental approach that uses time series data to assess the introduced public health interventions at population level [28–32]. We used autoregressive models to examine both immediate change in level of prescribing and a slope change in quarterly medication use in relation to COVID-19 pandemic measures [33]. These models allowed examination of the changes in medication prescribing while accounting for auto-correlation between consecutive quarterly observations and seasonality [28]. In all analyses, we assessed autocorrelation by visual inspection of the full and partial autocorrelation function plots and calculation of the Durbin-Watson statistic. If an interruption (COVID-19) influences the medication utilization, then a change in level and/or slope should be detected between pre- and post- phases while accounting for any secular trend in the pre-pandemic period. Percentage change was examined, which is calculated as the relative change in the percentage of dispensing between the first quarter of 2020 and the second quarter of 2020. We conducted sensitivity analyses by changing the intervention point of COVID-19 related restrictions in healthcare at the first quarter of 2020 instead of second quarter of 2020. Analyses were conducted using SAS, version 9.4 (SAS Institute, Inc). P-value <0.05 was used as the threshold for statistical significance.

### Ethical consideration and informed consent

This study has been approved by the University of Manitoba Health Research Ethics Board (HREB #: H2020:335) and access to data was approved by the Health Information Privacy Committee of Manitoba Health (HIPC #: 2020/2021-33). Consent was not required as data are de-identified.

## Results

During the study period, the quarterly study population ranged from 1,353,485 to 1,411,630 Manitobans. Of the individuals included in the study, the mean age was 38 years (SD = 23.35) and 50.18% of the population were female. Over 60% of the included population were living in urban areas. The most common comorbidities were asthma (26.67%) and hypertension (20.64%), followed by diabetes (8.31%) and coronary artery diseases (4.51%) **(Table 1).**

**Table 1:** Characteristics of the included Manitoba population from July 2016 to March 2021.

Characteristics^a^	2016	2017	2018	2019	2020	2021
**Age**, mean (SD)	38.12 (23.16)	37.4 (23.24)	37.94 (23.31)	38.21 (23.41)	38.44 (23.49)	39.20 (23.48)
**Age**, n (%)						
<65 years	1,167,490 (85.22)	1,198,939 (85.35)	1,205,512 (85.04)	1,200,198 (84.57)	1,197,346 (84.08)	1,174,693 (83.22)
≥ 65years	202,418 (14.78)	205,736 (14.65)	212,032 (14.96)	219,038 (15.43)	226,720 (15.92)	236,937 (16.78)
**Sex**, n (%)						
Male	682,575 (49.83)	701,068 (49.91)	707,052 (49.88)	706,998 (49.82)	708,856 (49.78)	701,912 (49.72)
Female	687,333 (50.17)	703,607 (50.09)	710,492 (50.12)	712,238 (50.18)	715,210 (50.22)	709,715 (50.28)
**Income**, n (%)						
Q1 (Lowest income)	275,346 (20.1)	282,166 (20.09)	289,561 (20.43)	288,167 (20.3)	283,879 (19.93)	276,666 (19.6)
Q2	271,027 (19.78)	283,893 (20.21)	280,021 (19.75)	281,296 (19.82)	282,072 (19.81)	278,506 (19.73)
Q3	269,042 (19.64)	277,359 (19.75)	282,568 (19.93)	282,488 (19.90)	282,698 (19.85)	280,136 (19.84)
Q4	269,461 (19.67)	276,312 (19.67)	279,621 (19.73)	280,741 (19.78)	282,430 (19.83)	281,109 (19.91)
Q5 (Highest income)	273,573 (19.97)	273,386 (19.46)	274,352 (19.35)	275,571 (19.42)	280,815 (19.72)	280,597 (19.88)
Unknown	11,459 (0.84)	11,559 (0.82)	11,421 (0.81)	10,973 (0.77)	12,172 (0.85)	14,616 (1.04)
**Residence**, n (%)						
Rural	523,509 (38.21)	532,981 (37.94)	537,556 (37.92)	541,255 (38.14)	546,970 (38.41)	545,329 (38.63)
Urban	846,399 (61.79)	871,694 (62.06)	879,988 (62.08)	877,981 (61.86)	877,096 (61.59)	866,301 (61.37)
**Comorbidities**^b^ n (%)						
Asthma	375,122 (24.27)	387,446 (25.06)	408,806 (26.44)	428,607 (27.73)	441,673 (28.57)	431,954 (27.94)
CAD	62,974 (4.07)	65,238 (4.22)	68,682 (4.44)	71,709 (4.64)	74,270 (4.80)	75,176 (4.86)
CHF^c^	17,515 (1.13)	19,033 (1.23)	22,266 (1.44)	25,547 (1.65)	28,630 (1.85)	31,389 (2.03)
Diabetes	112,495 (7.28)	116,522 (7.54)	124,357 (8.04)	132,368 (8.56)	139,736 (9.04)	145,707 (9.43)
Hypertension^d^	284,702 (18.42)	291,956 (18.89)	307,091 (19.86)	328,189 (21.23)	344,116 (22.26)	358,196 (23.17)

^a^ Characteristics: Patients counted at the first record in each year.

^b^ Comorbidities: Five-year look back period was used to calculate case definition percentages.

^c^ CHF; Congestive Heart Failure for those aged 40 and older.

^d^ Hypertension; for those aged 19 and older.

CAD: Coronary artery diseases.

**Fig 1:**
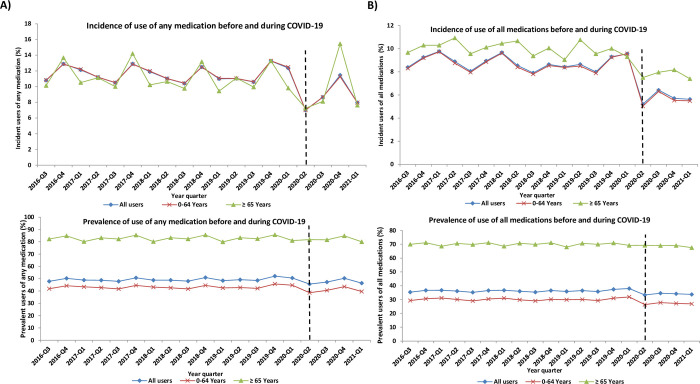
Incidence and Prevalence of (A) any medication and (B) all medications use stratified by age in Manitoba from Q3-2016 until Q1-2021.

**Fig 2:**
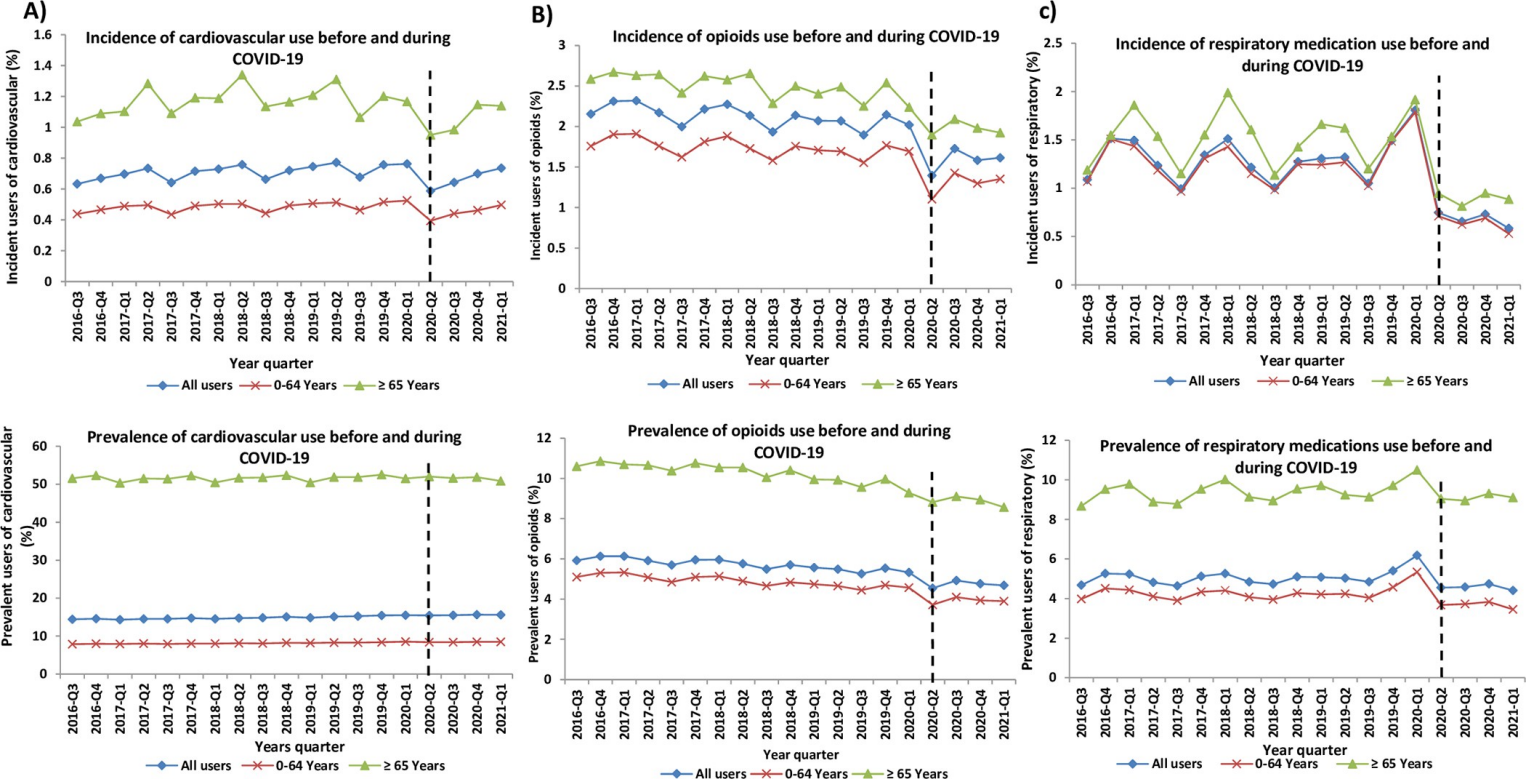
Incidence and Prevalence of (A) Cardiovascular, (B) Opioids and (C) respiratory medications use stratified by age in Manitoba from Q3-2016 until Q1-2021.

**Fig 3:**
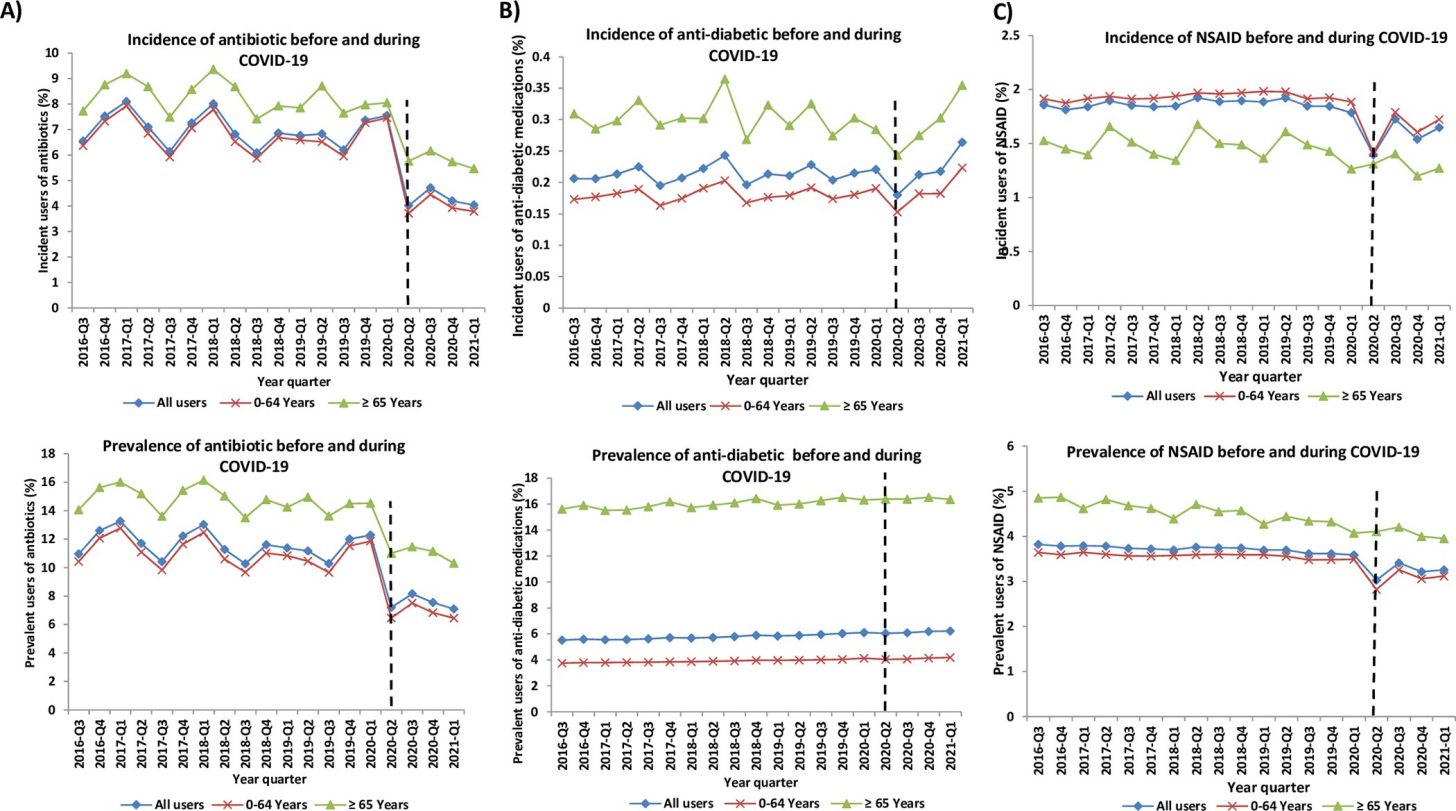
Incidence and Prevalence of (A) Antibiotic, (B) Anti-diabetic and (C) Non-steroidal anti-inflammatory drugs use stratified by age in Manitoba from Q3-2016 until Q1-2021.

During Period 2, i.e., the COVID-19 pandemic, from Q2-2020 to Q1-2021, the most used medications were cardiovascular drugs (15.6%), statins (9.9%), PPI (8.2%) followed by antibiotics and antidiabetics. **Figs 1–3** and **S1–S4 Figs** demonstrate the incidence and prevalence of different medication classes, by age group. The quarterly utilization of prescribed NSAIDs was the lowest among incident users of age ≥65 years in comparison to those of age <65 years old and all incident users **(Fig 3C)**. From 2016 to the first quarter of 2021, the overall number of antibiotic prescriptions showed a downward trend among all users. After the onset of the pandemic restrictions, prevalent users <65 years and ≥65 years old dropped by 5.4% and 3.5%, respectively **(Fig 3A)**. We observed a decreasing trend for corticosteroids (incident and prevalent) use among different age groups shortly after COVID-19 restrictions were implemented, followed by a steady trend increase.

Overall, selected medication classes in our database experienced a 45.55% and 12.17% relative quarterly decline in utilization immediately after the implementation of COVID-19 restrictions among both incident (3.1323% reduction; p = 0.002) and prevalent (3.0627% reduction; p = 0.005) users, respectively **(Table 2).** However, the overall change in slope in the quarterly incidence and prevalence of all medications use did not change significantly.

**Table 2:** Interrupted time series using autoregressive models summarizing the association between COVID-19 pandemic measures and quarterly medication utilization among incident and prevalent users in Manitoba, Canada.

Prescription Medication Class	Incident users^a^							Prevalent users^b^						
	Change in level^c^			*Pre-COVID slope*	*During COVID slope*	Change in slope^d^		Change in level^c^			*Pre-COVID slope*	*During COVID slope*	Change in slope^d^	
	*Percentage Change*	*Parameter estimate*	*P value*			*Parameter estimate*	*P value*	*Percentage change*	*Parameter estimate*	*P value*			*Parameter estimate*	*P value*
All Drugs	**-45.55%**	**-3.1323**	**0.002**	-0.0098	0.0569	0.0666	0.817	**-12.17%**	**-3.0627**	**0.005**	0.0711	0.0781	0.0070	0.983
Any Drug	**-43.25%**	**-4.3665**	**0.017**	0.0148	0.5592	0.5444	0.344	-9.75%	-4.0170	0.053	0.1115	0.5303	0.4188	0.531
Alpha-1 blockers	**-12.98%**	**-0.0356**	**0.001**	0.0013	0.0149	**0.0136**	**< .001**	0.48%	-0.0209	0.400	0.0183	0.0334	0.0151	0.087
Antibiotics	**-46.83%**	**-2.5272**	**0.008**	-0.0191	-0.0432	-0.0242	0.932	**-41.45%**	**-3.5961**	**0.010**	-0.0441	-0.0911	-0.0470	0.911
Antidiabetics	**-18.41%**	**-0.0634**	**0.002**	0.0005	0.0257	**0.0251**	**0.001**	-0.89%	-0.0663	0.233	0.0399	0.0633	0.0234	0.218
Antivirals	-8.69%	-0.0023	0.192	0.0003	-0.0001	-0.0004	0.536	-2.18%	-0.0030	0.371	0.0020	0.0018	-0.0002	0.847
Bisphosphonates	6.30%	-0.0026	0.809	-0.0003	0.0002	0.0005	0.893	3.18%	-0.0154	0.505	0.0038	0.0003	-0.0035	0.656
Cardiovascular	**-23.05%**	**-0.2138**	**< .001**	0.0062	0.0501	**0.0439**	**0.014**	-0.31%	0.0292	0.869	0.0788	0.0589	-0.0200	0.740
Chemotherapy	-8.49%	-0.0109	0.138	0.0017	0.0019	0.0003	0.898	-3.56%	-0.0184	0.061	0.0064	0.0085	0.0021	0.509
Corticosteroids	-32.13%	-0.1377	0.270	0.0021	-0.0149	-0.0170	0.685	-22.26%	-0.2219	0.085	0.0055	-0.0355	-0.0411	0.333
Hydroxychloroquine	-38.18%	-0.0034	0.435	0.0004	-0.0005	-0.0008	0.568	-4.18%	0.0062	0.473	0.0036	0.0028	-0.0007	0.802
Immunostimulant	19.25%	0.0008	0.549	-0.0001	-0.0001	0.0001	0.885	0.24%	-0.0004	0.757	-0.0003	0.0002	0.0005	0.333
Immunosuppressants	-12.82%	-0.0052	0.167	0.0003	0.0015	0.0012	0.329	-0.49%	-0.0141	0.062	0.0118	0.0145	0.0028	0.266
Neuraminidase inhibitors	-97.59%	-0.1702	0.266	0.0122	-0.0021	-0.0143	0.781	-97.24%	-0.1741	0.265	0.0126	-0.0024	-0.0151	0.774
NSAIDs	**-21.80%**	**-0.4289**	**< .001**	0.0003	0.05652	0.0562	0.081	**-15.56%**	**-0.5175**	**< .001**	-0.0139	0.0491	0.0630	0.069
Opioids	**-30.98%**	**-0.5546**	**0.002**	-0.0169	0.0517	0.0687	0.200	**-14.79%**	**-0.6824**	**0.003**	-0.0538	0.0265	0.0803	0.242
PPI	-14.35%	-0.2270	0.218	0.0272	0.0457	0.0185	0.763	-2.42%	0.1131	0.733	0.0704	0.1334	0.0630	0.579
Respiratory	**-58.62%**	**-0.6101**	**0.048**	0.0118	-0.0413	-0.0531	0.592	-26.39%	-0.7164	0.126	0.0390	-0.0283	-0.0673	0.663
Statins	**-27.89%**	**-0.1114**	**0.005**	0.0012	0.0385	**0.0372**	**0.006**	0.09%	0.0572	0.692	0.0584	0.0904	0.0321	0.518
Thyroid	**-26.66%**	**-0.0456**	**0.003**	-0.0018	0.0050	0.0068	0.136	-1.12%	0.0262	0.698	0.0172	-0.0164	-0.0336	0.159

^a^ Incident users: defined as people who had not been dispensed the examined medication of interest in the previous 4 quarters to their first dispensation

^b^ Prevalent users defined as people who used the medication within each quarter

^c^ Change in level is the immediate level change in the quarterly medication use after the implementation of COVID-19 restrictions

^d^ Change in slope is the difference in slope in the quarterly medication use between pre-pandemic period and pandemic period

^e^ Percentage change = (percentage in Q2 2020 –Percentage in Q1 2020)/ Percentage in Q1 2020

* 100

NSAIDS: Non-steroidal anti-inflammatory drugs; PPI: Pronton Pump Inhibitors.

We observed a 23.05% relative reduction in quarterly cardiovascular utilization among incident users (0.2138% reduction; p<0.001) after the onset of pandemic measures. There was also a significant increase in slope during COVID-19 among incident cardiovascular users (0.0439% increase quarterly; p = 0.014) compared to pre-COVID-19 period. In contrast, a non-significant drop in slope (0.31% decrease quarterly; p = 0.869) was found among the quarterly cardiovascular prevalent users immediately after the implementation of COVID-19 measures **(Table 2).**

After the onset of the pandemic restrictions, we noted a relative drop by 27.89% in the quarterly incidence of statins use (0.1114% reduction; p<0.005). Additionally, there was a significant increase in slope (0.0372% increase quarterly; p = 0.006) during the pandemic among incident statin users compared to pre-pandemic period. Nevertheless, the pandemic restrictions led to non-significant changes among prevalent statin users. Moreover, we observed no significant changes among both incident and prevalent users of PPI during the pandemic period **(Table 2).**

Among incident users, we found a 18.41% relative drop in quarterly antidiabetics use (0.0634% reduction; p = 0.002) rapidly after the implementation of COVID-19 mitigations measures during the first quarter of 2020. There was a non-significant increase in the quarterly prevalent use of antidiabetics, increasing from 5.5% during the third quarter of 2016 to 6.23% during the first quarter of 2021 (0.0663% reduction; p = 0.218). Additionally, we observed a significant increase in slope (0.0251% increase quarterly; p = 0.001) among incident antidiabetics use during the pandemic period compared to before pandemic period **(Table 2).**

After the implementation of COVID-19 restrictions, there was 30.98% and 14.79% relative drop in the quarterly incidence (0.5546% reduction; p = 0.002) and prevalence (0.6824% reduction; p = 0.003) of opioid use, respectively. However, a non-significant rise in slope was noted among both the incident (0.0687% increase quarterly; p = 0.200) and prevalent users (0.0803% increase quarterly; p = 0.242) during COVID-19 period. Throughout the study period (2016–2021), the overall consumption of opioids showed a downward trend in both prevalence and incident users.

COVID-19 mitigation measures led to a significant decline in the quarterly prescriptions for incident users of NSAIDs (0.4289% reduction; p<0.001), antibiotics (2.5272% reduction; p = 0.008), and respiratory medications (0.6101% reduction; p = 0.048). Moreover, we noted a 15.56% and 41.45% relative drop among the quarterly NSAIDs (0.5175% reduction; p<0.001) and antibiotics (3.5961% reduction; p = 0.010) prevalent users, respectively. However, there was no significant change in the slope observed among incidence and prevalence users for these medications. Furthermore, there was no statistically significant change in slope and change in percentage among the incidence and prevalence use of corticosteroids and antivirals during the pandemic period.

Immediately after the onset of the restrictions, we noted a significant decrease among incident users of thyroid medications (0.0456% reduction; p = 0.003) and alpha-1 adrenergic receptor blocker (0.0356% reduction; p = 0.001), respectively **(Table 2)**. However, the pandemic restrictions led to a non-significant change among incident and prevalent users of these medication classes: neuraminidase inhibitors, immunosuppressants, immunostimulants, hydroxychloroquine, and chemotherapeutic medication **(S1 and S2 Figs)**. The sensitivity analyses showed similar results to those of the main analyses.

## Discussion

### Main findings

This study quantified the incidence and prevalence of several drug classes before and during the pandemic period of COVID-19 in Manitoba, Canada. When considered as aggregate use of overall drugs- both within incident and prevalent users—we observed a statistically significant immediate decline in overall utilization. We observed that the use of most medication classes was disrupted following the COVID-19 restrictions, with statistically significant changes in 9 of the 18 medication classes examined. Furthermore, the difference in the percentage of disruption of all medications use was higher among young individuals compared to those ≥65 years old. During this time, the reduction in use may have resulted from pandemic-driven changes to the health care system, including the cancellation of non-urgent in-person appointments and procedures and stresses to the drug supply [34]. In addition, based on media anecdotes, the stockpiling of medications (where prescriptions would be filled less frequently, in higher volumes) in response to public concerns of drug shortages has the potential to result in irregular and atypical utilization patterns [16]. Shortly after mitigation measures were implemented, medication utilization began to stabilize, reflecting a transition to telehealth, stabilization of drug supply, and the restriction and subsequent relaxation of medication days’ supply limits that patients could obtain from their pharmacies [9, 10].

Three commonly acute drug classes (antibiotics, NSAIDs, and opioids) experienced a sudden significant decline in incident and prevalent use when mitigation measures were enacted. Antibiotics use in Manitoba has declined since 2016, with similar trends among prevalent users of all ages during the pandemic. This trend suggests that public health measures like social distancing and wearing masks resulted in a significant decline in antibiotics incident users, but no significant change in slope in neither incident nor prevalent users was observed. It is likely that due to these measures and cancellation of non-urgent procedures, fewer individuals developed microbial infections. It is possible that the changes to health care access have hindered individuals from seeking out medical attention, promoting the “wait and see” method, therefore, limiting potential inappropriate antimicrobial prescribing for self-limiting conditions. Several studies show similar patterns, as nursing home in Ontario observed no significant changes in slope or level, and in the US, two studies observed a decline in antimicrobial use [17, 35, 36]. Likewise, in Italy, antimicrobial use decreased during the pandemic; however, an increase in azithromycin was seen early on, likely due to early theories of its efficacy in treating COVID-19 [37]. In Australia, a 16% decrease in antimicrobial use in dental prescriptions then a 20% increase when restrictions eased was observed [38]. The prescriptions of both opioids and NSAIDs experienced a significant change in level for incident users upon implementing COVID-19 mitigation measures in Manitoba. The use of opioids has decreased since 2016 in both incident and prevalent users. COVID-19 delayed non-urgent procedures and dental visits along with opioid stewardship measures becoming more common, both factors are important considerations for the changes in analgesic use during the study period [39]. Similarly, weekly community dispensing of opioids in Ontario decreased after the onset of the pandemic [40]. In contrast, nursing home residents in both Ontario and Michigan showed a significant increase in slope in opioid dispensing [17, 41]. Mian *et al*. observed an 18% decrease in opioid analgesics and a 41% decrease in NSAIDs dental prescriptions in Australia in April 2020 [38]. However, in June 2020, increases in tramadol, oxycodone, and NSAIDs (46%, 73%, 59%) were observed in comparison to the previous year [38].

Among incident users for chronic medication classes, the most disrupted classes were cardiovascular medications, antidiabetics, and respiratory medications. Cardiovascular medications were the most common drug class filled in Manitoba. These medications experienced a 23.05% drop in incident use during the first quarter of 2020; however, incident users experienced a non-significant change in slope. Drug shortage concerns with select angiotensin receptor blockers (ARBs) (i.e., valsartan) and early concerns that angiotensin-converting enzyme inhibitors and ARBs could potentially worsen COVID-19 patient outcomes should be noted. These factors may have altered prescribing patterns or patient adherence at the beginning of the pandemic, followed by the observed rebound effect. Nursing home residents taking ACE inhibitors or ARBs in Ontario experienced non-significant changes in level and slope for their residents and, in the USA, Vaduganathan *et al*. observed a non-significant decline in ACE inhibitors and ARBs use effect [17, 36, 42]. In addition, nursing home residents from Michigan, USA experienced no change in diuretic use [41]. Of note, the prescription of statins experienced a significant drop among Manitoban incident users immediately after the onset of the pandemic restrictions. Whaley *et al*. reported that individuals in the USA had a 2.8% decrease in statins use effect while Esposti *et al*. found an increase in missed refills of statins by 38.6% during the lockdown period in Italy [14, 43]. Incident users of antidiabetic medications have experienced a significant increase in slope, which could be attributed to the rebound effect of this medication use. Antidiabetic medications have increased in quarterly use from 5.5% in 2016 to 6.2% in 2021. In contrast, Whaley *et al*. observed that the use of antidiabetics in the USA has decreased by 2.3% [14]. Respiratory medications experienced a significant decline in level for incident users. In contrast, Whaley *et al*. observed an 11.1% increase in asthma medications [14].

PPI prescriptions have increased in both incident and prevalent users since 2016, however, COVID-19 restrictions have resulted in a sudden non-significant decline followed by a rebound increase. This decline may be due to concerns about an increased likelihood of COVID-19 infection following PPI administration, which may have influenced prescribing patterns [44, 45]. In contrast, long-term care in the USA reported a 59% increase in famotidine prescriptions in April 2020 compared to 2019 [46]. The use of thyroid medications experienced a significant decrease by 26.66% among incident users in Manitoba. Similarly, between 2019 and 2020, Inoue *et al*. found a significant decline by 17% in the monthly follow-up visits among patients treated with levothyroxine [47]. Antiviral use in Manitoba did not significantly change; however, reports from Ontario and a worldwide study showed a 49.3% and 85% respective decrease in direct-acting antivirals during the pandemic [15, 48]. Hydroxychloroquine use among Manitobans did not significantly change during the pandemic unlike USA counterparts in which a surge of hydroxychloroquine prescriptions was observed, perhaps in part due to media and political attention, as 214.1% increase were seen between March 15–21, 2020, in comparison to 2019 [36].

COVID-19 restrictions served as an interruption to drug utilization worldwide. Therefore, future drug utilization studies using prescription drug databases should carefully consider the COVID-19 period. Additionally, understanding the changes will guide policymakers and health care providers during future health outbreaks to ensure best practices—like continuing appointments via telehealth and alternate formularies for drug shortages—are developed and implemented promptly.

Our study has important strengths: we used prescriptions data that covered the entire Manitoba population without restrictions of age, sex, income, or insurance coverage, providing a generalizable overview of pandemic-related changes in medication prescribing, unlike many other databases. Nevertheless, our study has limitations. First, we did not correlate the observed changes in medication use with the COVID-19 infection rates. However, the number of COVID-19 infected patients during the first wave was extremely low in Manitoba (<30 cases), so this limitation would have a negligible impact on the results of the study. Second, we included the prescriptions dispensed to identify medication use, which may not reflect the actual consumption of such medications. Third, our study did not investigate the prescribing indications, this could be an area for future investigations. Furthermore, although there was a significant change in slope in some medication classes, the persistent effect the pandemic had on medication use remains uncertain since our study included 4 quarters after the onset of the pandemic. Additional research is warranted to fully understand the changes in the prescription utilization. Future research should investigate whether such changes in drug utilization were associated with potential adverse effects on patient outcomes.

## Conclusions

COVID-19 mitigation measures led to a temporary but significant decrease in prescription drugs utilization, especially among incident users. Our findings highlight the effects COVID-19 restrictions have had on prescribing practices in the Canadian province of Manitoba. As newer quarterly data becomes available, further studies on how drug utilization and supply respond to the easing of restrictions, return of in-person health care visits, and future COVID-19 variants should be explored. Additionally, monitoring the prescribing trends for incident and prevalent users of antimicrobials and opioids is essential in promoting patient safety and guiding future prescribing practices.

## Supporting information

S1 Fig Incidence and Prevalence of (A) Chemotherapy, (B) immunostimulants, and (C) immunosuppressants use stratified by age in Manitoba from Q3-2016 until Q1-2021.
(TIF)

S2 Fig Incidence and Prevalence of (A) Hydroxychloroquine, (B) Corticosteroids, and (C) neuro-inhibitory, and stratified by age in Manitoba from Q3-2016 until Q1-2021.
(TIF)

S3 Fig Incidence and Prevalence of (A) Statin, (B) Alpha-1 adrenergic receptor blocker and (C) proton pump inhibitors use in Manitoba from Q3-2016 until Q1-2021.
(TIF)

S4 Fig Incidence and Prevalence of (A) thyroid, (B) bisphosphonates, and (C) antiviral use in Manitoba from Q3-2016 until Q1-2021.
(TIF)

S1 Table List of prescribed medications available in the Canadian market in Manitoba investigated during July 1, 2016 to March 31, 2021. (DOCX)
