# Supplementary figures and images for: Drug utilization patterns before and during COVID-19 pandemic in Manitoba, Canada: A population-based study
Source: PLoS One. 2022 Nov 28;17(11):e0278072. doi: 10.1371/journal.pone.0278072 (PMC9704650; doi:10.1371/journal.pone.0278072)

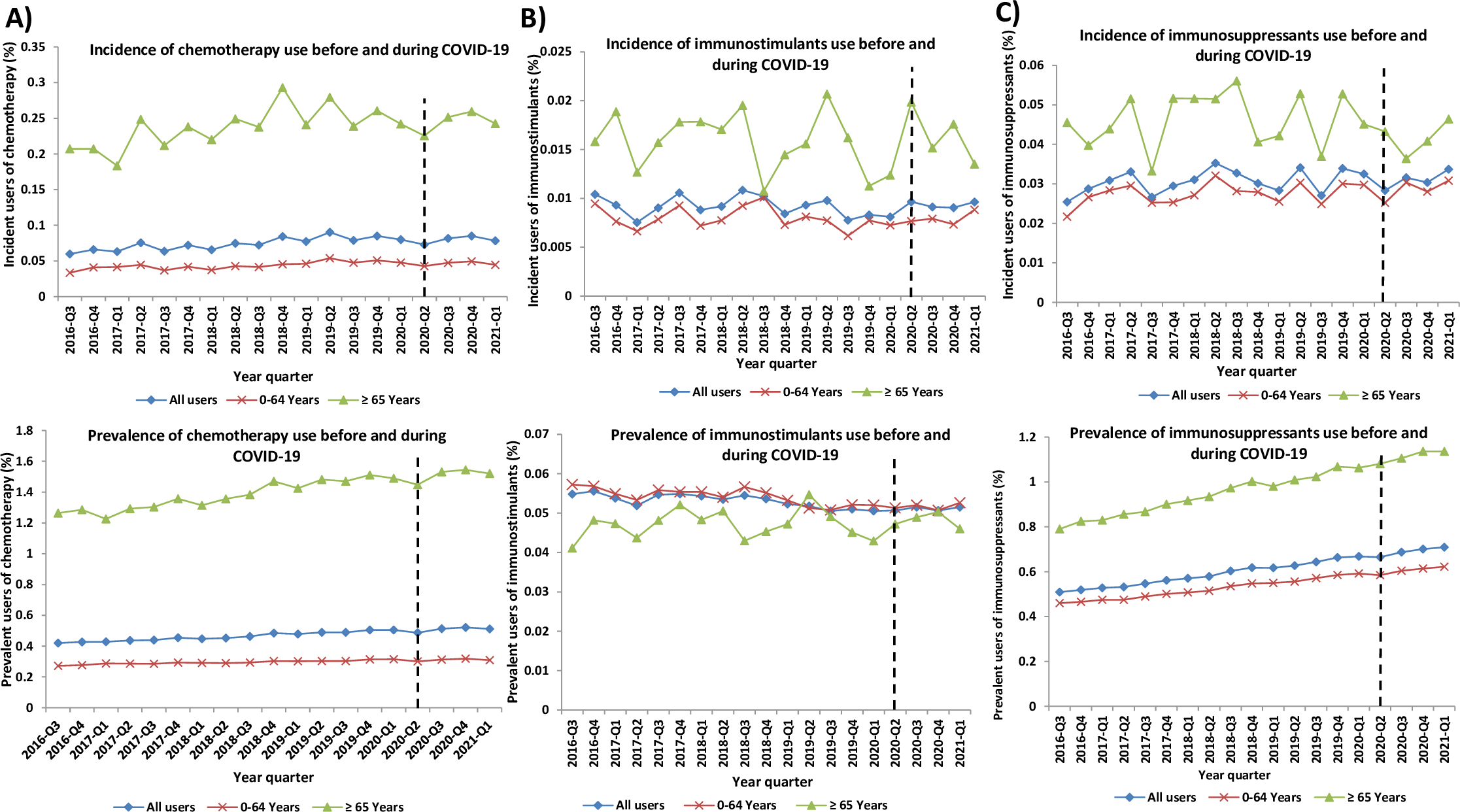

Supplement: S1 Fig — Incidence and Prevalence of (A) Chemotherapy, (B) immunostimulants, and (C) immunosuppressants use stratified by age in Manitoba from Q3-2016 until Q1-2021. (TIF) [file pone.0278072.s001.tif]

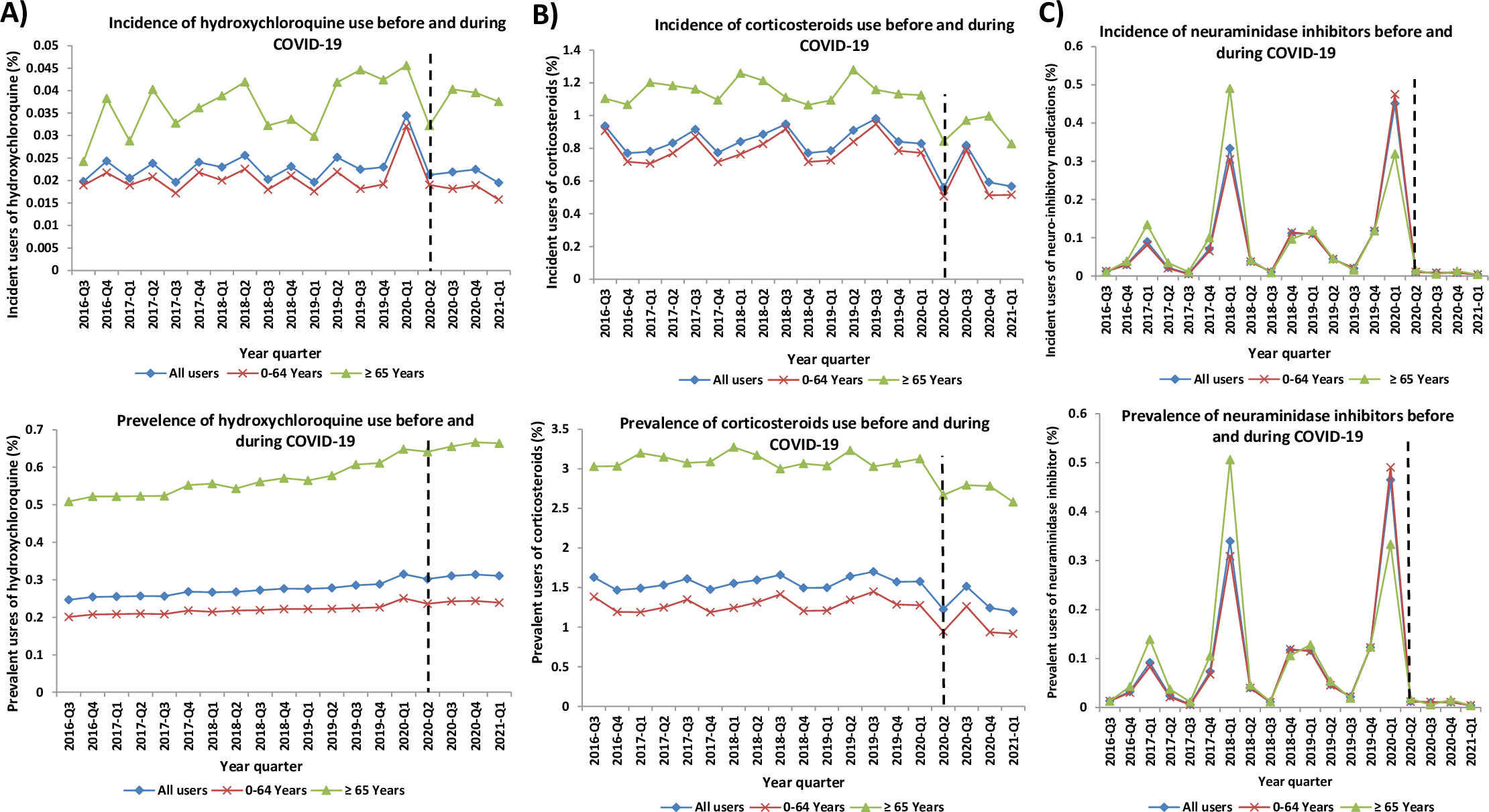

Supplement: S2 Fig — Incidence and Prevalence of (A) Hydroxychloroquine, (B) Corticosteroids, and (C) neuro-inhibitory, and stratified by age in Manitoba from Q3-2016 until Q1-2021. (TIF) [file pone.0278072.s002.tif]

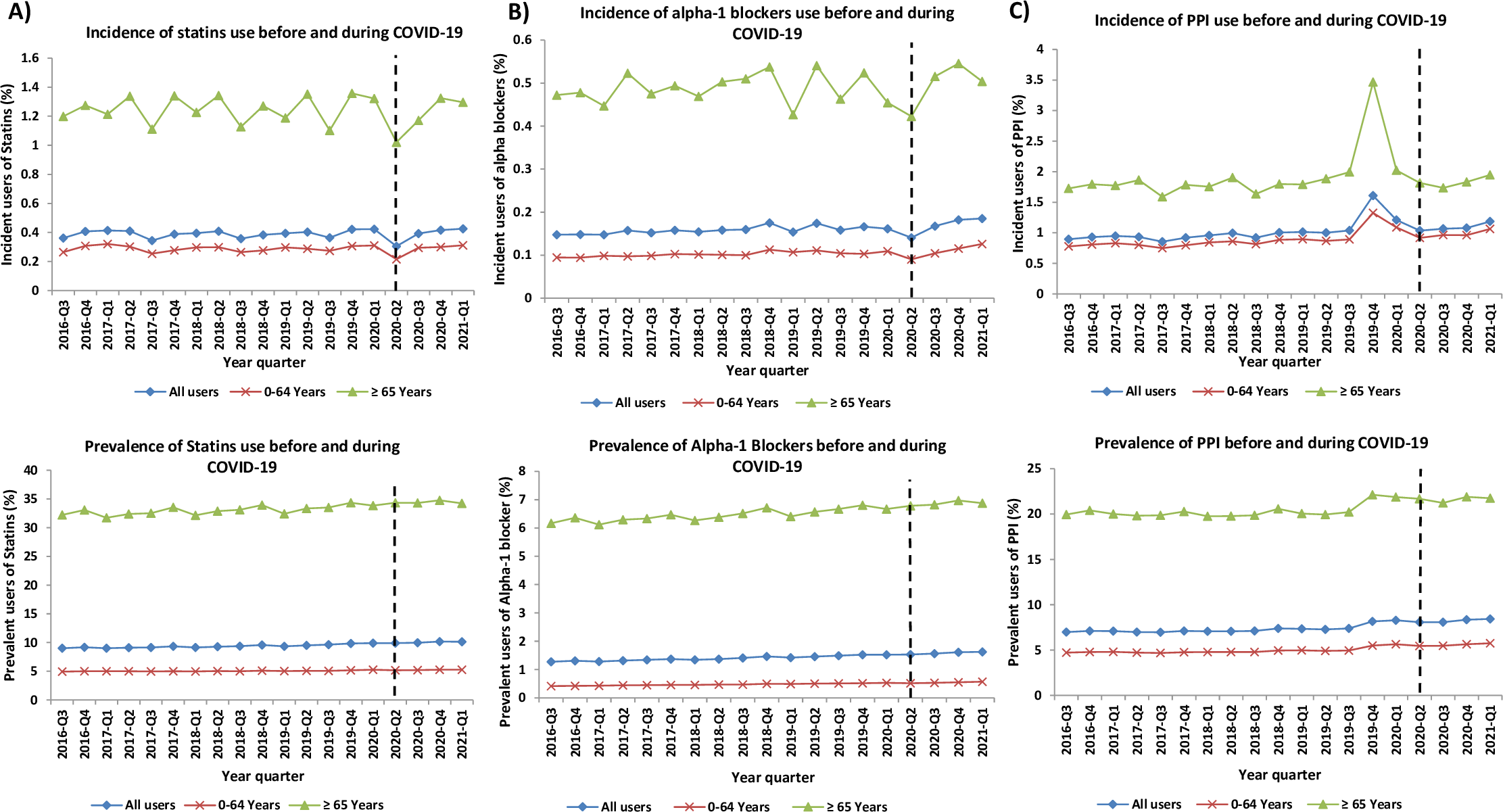

Supplement: S3 Fig — Incidence and Prevalence of (A) Statin, (B) Alpha-1 adrenergic receptor blocker and (C) proton pump inhibitors use in Manitoba from Q3-2016 until Q1-2021. (TIF) [file pone.0278072.s003.tif]

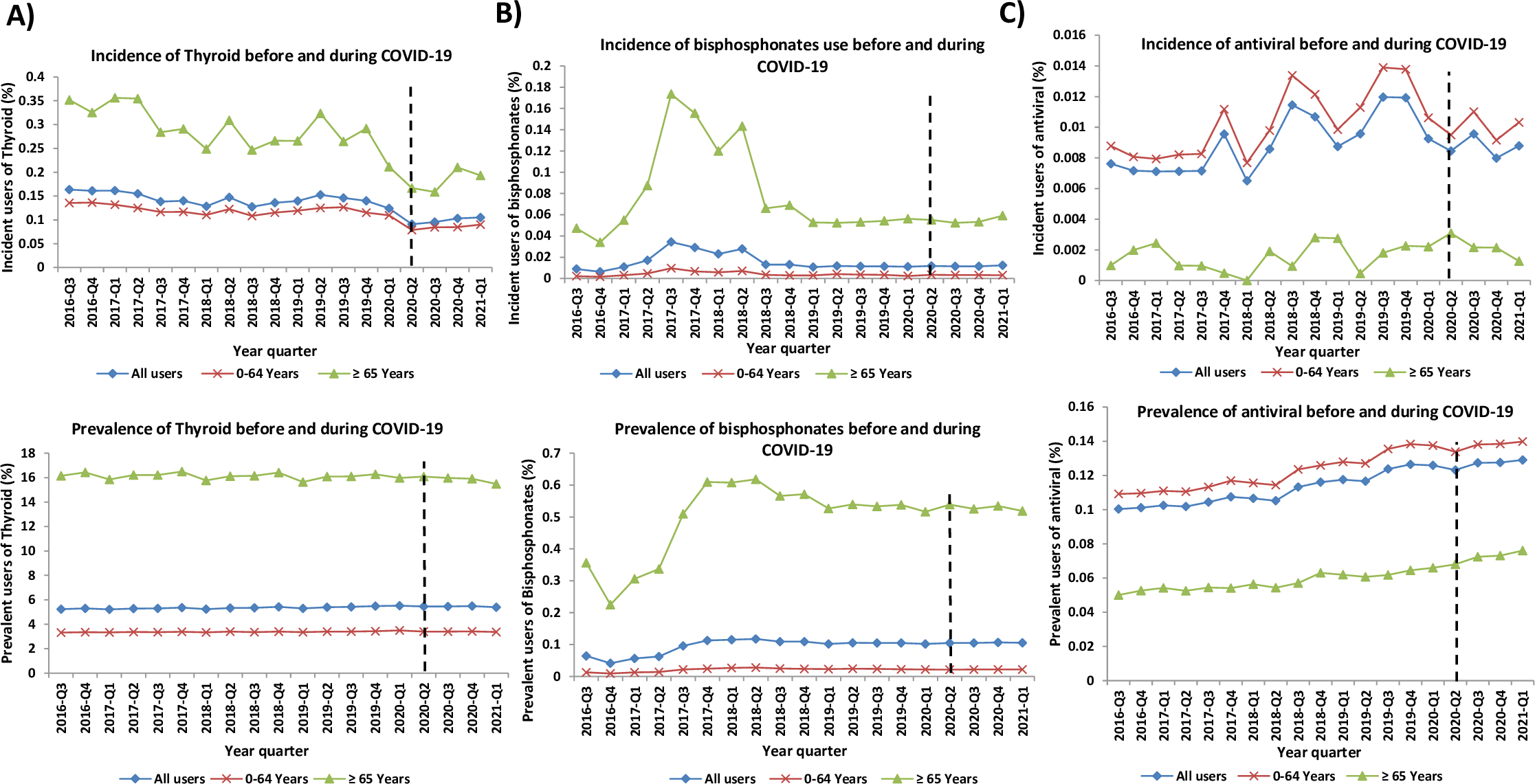

Supplement: S4 Fig — Incidence and Prevalence of (A) thyroid, (B) bisphosphonates, and (C) antiviral use in Manitoba from Q3-2016 until Q1-2021. (TIF) [file pone.0278072.s004.tif]
