# Supplementary material for: Drug utilization patterns before and during COVID-19 pandemic in Manitoba, Canada: A population-based study
Source: PLoS One. 2022 Nov 28;17(11):e0278072. doi: 10.1371/journal.pone.0278072 (PMC9704650; doi:10.1371/journal.pone.0278072)
Supplement: S1 Table — (DOCX) [file pone.0278072.s005.docx]

**S1 Table.** List of prescribed medications available in the Canadian market in Manitoba investigated during July 1, 2016 to March 31, 2021.

| **Medications** | **Dosage form** |
| --- | --- |
| 1. **Cardiovascular Medications:** | |
| ***Beta-blockers*** | |
| Acebutolol hydrochloride | Tablet |
| Atenolol | Tablet |
| Bisoprolol fumarate | Tablet |
| Carvedilol | Tablet |
| Esmolol hydrochloride | Liquid/solution |
| Labetalol hydrochloride | Tablet/Liquid/solution |
| Metoprolol succinate | Tablet sustained release |
| Metoprolol tartrate | Solution/Tablet |
| Nadolol | Tablet |
| Nebivolol hydrochloride | Tablet |
| Oxprenolol hydrochloride | Tablet |
| Pindolol | Tablet |
| ***Angiotensin-converting enzyme inhibitor*** | |
| Benazepril hydrochloride | Tablet |
| Captopril | Tablet |
| Cilazapril | Tablet |
| Enalapril maleate | Tablet |
| Enalaprilat | Solution |
| Fosinopril sodium | Tablet |
| Lisinopril | Tablet |
| Perindopril arginine | Tablet |
| Perindopril erbumine | Tablet |
| Quinapril | Tablet |
| Ramipril | Capsule/Tablet |
| Trandolapril | Capsule |
| ***Angiotensin-receptor blockers*** | |
| Azilsartan medoxomil | Tablet |
| Candesartan cilexetil | Tablet |
| Eprosartan | Tablet |
| Irbesartan | Tablet |
| Losartan potassium | Tablet |
| Olmesartan medoxomil | Tablet |
| Telmisartan | Tablet |
| Valsartan | Capsule/Tablet |
| ***Calcium Channel blockers*** | |
| Amlodipine | Solution/Tablet/Emulsion |
| Clevidipine | Emulsion |
| Diltiazem HCL | Capsule/Tablet/Powder for solution |
| Felodipine | Tablet |
| Nicardipine HCL | Capsule/Liquid |
| Nifedipine | Capsule/Tablet |
| Nimodipine | Capsule/Liquid |
| Verapamil HCL | Tablet/Capsule/Liquid |
| ***Diuretics*** | |
| Amiloride hydrochloride | Tablet |
| Bendroflumethiazide | Tablet |
| Bumetanide | Tablet |
| Chlorthalidone | Tablet |
| Eplerenone | Tablet |
| Ethacrynic acid | Tablet/Powder for solution |
| Furosemide | Tablet/Liquid/solution |
| Hydrochlorothiazide | Tablet |
| Indapamide | Tablet |
| Metolazone | Tablet |
| Pamabrom | Capsule/Tablet |
| Spironolactone | Tablet |
| Tolvaptan | Tablet |
| Torsemide | Tablet/Liquid |
| Triamterene | Tablet |
| ***Antihypertensive combinations*** | |
| Aliskiren /hydrochlorothiazide | Tablet |
| Aliskiren fumarate /amlodipine | Tablet |
| Amiloride HCL/ hydrochlorothiazide | Tablet |
| Amlodipine /atorvastatin | Tablet |
| Amlodipine /perindopril arginine | Tablet |
| Amlodipine /telmisartan | Tablet |
| Atenolol/chlorthalidone | Tablet |
| Azilsartan/chlorthalidone | Tablet |
| Bendroflumethiazide/nadolol | Tablet |
| Candesartan /hydrochlorothiazide | Tablet |
| Chlorothiazide/methyldopa | Tablet |
| Chlorthalidone/clonidine HCL | Tablet |
| Cilazapril/hydrochlorothiazide | Tablet |
| Enalapril maleate / hydrochlorothiazide | Tablet |
| Eprosartan/hydrochlorothiazide | Tablet |
| Felodipine /ramipril | Tablet |
| Hydralazine hydrochloride/ hydrochlorothiazide/reserpine | Tablet |
| Hydrochlorothiazide /irbesartan | Tablet |
| Hydrochlorothiazide/lisinopril | Tablet |
| Hydrochlorothiazide/losartan | Tablet |
| Hydrochlorothiazide /olmesartan medoxomil | Tablet |
| Hydrochlorothiazide/ quinapril | Tablet |
| Hydrochlorothiazide /ramipril | Tablet |
| Hydrochlorothiazide/telmisartan | Tablet |
| Hydrochlorothiazide /valsartan | Tablet |
| Hydrochlorothiazide /methyldopa | Tablet |
| Hydrochlorothiazide/ reserpine | Tablet |
| Hydrochlorothiazide/spironolactone | Tablet |
| Hydrochlorothiazide /timolol | Tablet |
| Hydrochlorothiazide/triamterene | Tablet |
| Indapamide/perindopril | Tablet |
| Sacubitril/valsartan | Tablet |
| Trandolapril/verapamil HCL | Tablet |
| ***Blood thinners*** | |
| Apixaban | Tablet |
| Dabigatran | Capsule |
| Edoxaban | Tablet |
| Rivaroxaban | Tablet |
| Warfarin | |
| 1. ***Corticosteroids*** | |
| Betamethasone | Tablet |
| Cortisone acetate | Tablet |
| Dexamethasone | Elixir/Tablet |
| Fludrocortisone acetate | Tablet |
| Hydrocortisone | Tablet |
| Methylprednisolone | Tablet |
| Prednisolone | Tablet/Liquid/solution |
| Prednisone | Tablet |
| Triamcinolone | Tablet |
| 1. ***Antidiabetics*** | |
| Acarbose | Tablet |
| Acetohexamide | Tablet |
| Albiglutide | Powder for solution |
| Alogliptin | Tablet |
| Canagliflozin | Tablet |
| Chlorpropamide | Tablet |
| Dapagliflozin | Tablet |
| Dulaglutide | SOLUTION |
| Empagliflozin | Tablet |
| Ertugliflozin | Tablet |
| Exenatide | Powder for suspension/Solution/Suspension |
| Gliclazide | Tablet |
| Glimepiride | Tablet |
| Glipizide | Tablet |
| Glyburide | Tablet |
| Human insulin | Powder |
| Insulin Biosynthetic Human Br | Suspension |
| Insulin, Insulin zinc, Insulin aspart  Insulin degludec, Insulin glargine  Insulin isophane, Insulin lispro  Insulin NPH human DNA origin, Insulin Toronto | Liquid/suspension |
| Insulin human biosynthetic/insulin isophane human biosynthetic | Suspension |
| Linagliptin | Tablet |
| Linagliptin/metformin | Tablet |
| Liraglutide | Solution |
| Lixisenatide | Solution |
| Metformin HCL | Tablet |
| Metformin HCL/rosiglitazone | Tablet |
| Metformin HCL/saxagliptin | Tablet |
| Metformin HCL/sitagliptin | Tablet |
| Miglitol | Tablet |
| Nateglinide | Tablet |
| Pioglitazone | Tablet |
| Repaglinide | Tablet |
| Rosiglitazone | Tablet |
| Saxagliptin | Tablet |
| Semaglutide | Solution/Tablet |
| Sitagliptin | Tablet |
| Tolbutamide | Tablet |
| Troglitazone | Tablet |
| 1. ***Antimalarials*** | |
| Chloroquine | Tablet |
| Hydroxychloroquine | Tablet |
| 1. ***Antibiotics*** | |
| Amikacin | Solution/Liquid |
| Amoxicillin | Capsule/powder for solution/suspension |
| Amoxicillin/clavulanate potassium | Tablet/Capsule/Powder for suspension |
| Ampicillin | Tablet/Capsule/Powder for suspension |
| Azithromycin | Capsule/Tablet/powder for solution/suspension |
| Aztreonam | Powder for solution |
| Bacampicillin HCL | Tablet |
| Bacitracin | Powder for solution |
| Cefaclor | Tablet/Capsule/Powder |
| Cefadroxil | Capsule |
| Cefamandole | Powder |
| Cefazolin | Powder |
| Cefdinir | Suspension/Capsule |
| Cefepime | Powder |
| Cefixime | Powder/Tablet |
| Cefonicid sodium | Powder |
| Cefotaxime | Powder |
| Cefotetan | Powder |
| Cefoxitin | Powder |
| Cefpodoxime | Tablet |
| Cefprozil | Powder/Tablet |
| Ceftazidime | Powder |
| Ceftibuten | Powder/Capsule |
| Ceftizoxime | Powder |
| Ceftobiprole | Powder |
| Ceftriaxone | Powder/Solution |
| Cefuroxime | Powder/Tablet/Solution |
| Cephalexin | Powder/Tablet/Suspension/Capsule |
| Cephalothin | Powder |
| Chloramphenicol | Capsule/Powder |
| Cilastatin/imipenem | Powder |
| Ciprofloxacin | Powder/Tablet/Suspension/Solution |
| Clarithromycin | Tablet |
| Clavulanic acid /ticarcillin | Powder |
| Clindamycin | Granule/Capsule/Solution |
| Cloxacillin | Capsule/Powder |
| Colistin | Powder |
| Dalbavancin | Powder |
| Dalfopristin /quinupristin | Powder |
| Daptomycin | Powder |
| Demeclocycline HCl | Tablet |
| Doripenem | Powder |
| Doxycycline | Tablet/Capsule/Powder |
| Ertapenem | Powder |
| Erythromycin | Tablet/Capsule/Powder/Suspension |
| Fleroxacin | Tablet/Liquid |
| Floxacillin | Capsule/Powder/Liquid |
| Fosfomycin | Powder |
| Fusidate sodium | Tablet |
| Gatifloxacin | Tablet/Capsule/Liquid |
| Gemifloxacin | Tablet |
| Gentamicin /sodium chloride | Solution |
| Gentamicin | Liquid/Solution |
| Grepafloxacin | Tablet |
| Lefamulin | Solution/Tablet |
| Levofloxacin | Solution/Tablet |
| Linezolid | Powder/Solution/Tablet |
| Loracarbef | Powder |
| Meropenem | Powder |
| Methenamine Hippurate | Tablet |
| Metronidazole | Tablet/Capsule/Solution |
| Minocycline | Tablet/Capsule |
| Moxifloxacin | Tablet/Solution |
| Nafcillin | Powder |
| Nalidixic acid | Tablet |
| Neomycin sulfate | Tablet/Solution |
| Netilmicin sulfate | Solution |
| Nitrofurantoin | Tablet/Capsule |
| Norfloxacin | Tablet |
| Ofloxacin | Tablet/Liquid |
| Penicillin G | Powder/Tablet/Suspension |
| Penicillin V | Tablet/Suspension/Liquid |
| Piperacillin /tazobactam | Powder |
| Pivampicillin | Powder/Tablet |
| Pivmecillinam | Tablet |
| Polymyxin B | Powder |
| Spectinomycin | Powder |
| Spiramycin | Capsule |
| Sulfadiazine | Suspension/Tablet |
| Sulfamethoxazole/trimethoprim | Tablet/Suspension |
| Sulfapyridine | Tablet |
| Sulfisoxazole | Tablet |
| Tedizolid phosphate | Tablet/Powder |
| Telavancin | Powder |
| Tetracycline HCL | Tablet/Capsule/Liquid |
| Ticarcillin disodium | Powder |
| Tigecycline | Powder |
| Tobramycin | Capsule/Liquid/powder |
| Trimethoprim | Tablet |
| Trovafloxacin | Tablet/Liquid |
| Vancomycin | Powder/Solution/Liquid |
| 1. ***Antivirals*** | |
| ABACAVIR | Tablet/Solution |
| ADEFOVIR DIPIVOXIL | Tablet |
| AMPRENAVIR | Capsule/Liquid |
| ATAZANAVIR | Capsule/Tablet |
| BICTEGRAVIR/EMTRICITABINE /TENOFOVIR ALAFENAMIDE | Tablet |
| CABOTEGRAVIR/RILPIVIRINE | Suspension |
| DARUNAVIR | Tablet |
| DELAVIRDINE MESYLATE | Tablet |
| DIDANOSINE | Tablet/Capsule |
| DOLUTEGRAVIR/LAMIVUDINE | Tablet |
| DORAVIRINE | Tablet |
| EFAVIRENZ | Tablet/Capsule |
| EMTRICITABINE | Capsule |
| EMTRICITABINE/RILPIVIRINE/TENOFOVIR | Tablet/Capsule |
| ENFUVIRTIDE | Powder |
| ENTECAVIR | Solution/Tablet |
| ETRAVIRINE | Tablet |
| FOSAMPRENAVIR | Tablet/Suspension |
| INDINAVIR | Capsule |
| LAMIVUDINE | Solution/Tablet |
| LOPINAVIR/RITONAVIR | Tablet/Capsule/Solution |
| MARAVIROC | Tablet |
| NELFINAVIR | Tablet/Powder |
| NEVIRAPINE | Tablet |
| RILPIVIRINE | Tablet |
| RITONAVIR | Capsule/Solution |
| SAQUINAVIR | Tablet/Capsule |
| STAVUDINE | Capsule/Powder |
| TELBIVUDINE | Tablet |
| TENOFOVIR | Tablet |
| TIPRANAVIR | Capsule |
| ZALCITABINE | Tablet |
| ZIDOVUDINE | Liquid/Tablet/Capsule |
| 1. ***Neuraminidase Inhibitors*** | |
| OSELTAMIVIR | Capsule/Powder |
| PERAMIVIR | Solution |
| ZANAMIVIR | Powder |
| 1. ***Immunosuppressants*** | |
| ABATACEPT | Solution/Powder |
| ADALIMUMAB | Solution |
| ALEFACEPT | Powder |
| ALEMTUZUMAB | Solution |
| ANAKINRA | Solution |
| ANTI-THYMOCYTE GLOBULIN | Powder |
| APREMILAST | Tablet |
| AZATHIOPRINE | Tablet/Powder |
| BARICITINIB | Tablet |
| BASILIXIMAB | Powder |
| BELIMUMAB | Powder/Solution |
| BRODALUMAB | Solution |
| CANAKINUMAB | Powder/Solution |
| CERTOLIZUMAB | Solution |
| CLADRIBINE | Tablet |
| CYCLOSPORINE | Capsule/Liquid/Solution |
| DIMETHYL FUMARATE | Capsule |
| ECULIZUMAB | Solution |
| EFALIZUMAB | Powder |
| ETANERCEPT | Solution |
| EVEROLIMUS | Tablet/Capsule |
| FINGOLIMOD | Capsule |
| GOLIMUMAB | Solution |
| GUSELKUMAB | Solution |
| INFLIXIMAB | Powder |
| IXEKIZUMAB | Solution |
| LEFLUNOMIDE | Tablet |
| LENALIDOMIDE | Capsule |
| LYMPHOCYTE IMMUNOGLOBULIN ANTI-THYMOCYTE GLOBULIN | Liquid/Solution |
| METHOTREXATE | Solution/Tablet |
| MUROMONAB-CD3 | Solution |
| MYCOPHENOLATE MOFETIL | Powder/Capsule/Tablet |
| NATALIZUMAB | Solution |
| OCRELIZUMAB | Solution |
| PIRFENIDONE | Capsule/Tablet |
| POMALIDOMIDE | Capsule |
| RAVULIZUMAB | Solution |
| RISANKIZUMAB | Solution |
| SARILUMAB | Solution |
| SATRALIZUMAB | Solution |
| SECUKINUMAB | Powder/Solution |
| SILTUXIMAB | Powder |
| SIPONIMOD | Tablet |
| SIROLIMUS | Solution/Tablet |
| TACROLIMUS | Capsule/Tablet |
| TERIFLUNOMIDE | Tablet |
| THALIDOMIDE | Capsule |
| TOCILIZUMAB | Solution |
| TOFACITINIB | Tablet |
| UPADACITINIB | Tablet |
| USTEKINUMAB | Solution |
| VEDOLIZUMAB | Powder/Solution |
| 1. ***Immunostimulants*** | |
| ALDESLEUKIN | Powder |
| ANCESTIM | Powder |
| BACILLUS CALMETTE-GUERIN BCG | Powder/Liquid |
| BOCEPREVIR /PEGINTERFERON ALFA-2B | Capsule |
| FILGRASTIM | Solution |
| GLATIRAMER ACETATE | Powder/Solution |
| INTERFERONs | Powder/Solution/Capsule |
| MOLGRAMOSTIM | Powder |
| PEGFILGRASTIM | Solution |
| PEGINTERFERONs | Powder/Capsule/Liquid |
| PLERIXAFOR | Solution |
| SARGRAMOSTIM | Powder |

| 1. ***NSAIDs*** | |
| --- | --- |
| CELECOXIB | Capsule |
| DICLOFENAC SODIUM | Tablet/ SUPPOSITORY |
| ETODOLAC | Capsule |
| FENOPROFEN | Capsule |
| FLURBIPROFEN | TABLET |
| IBUPROFEN | Tablet / SUSPENSION/Capsule |
| INDOMETHACIN | Capsule / Suppository |
| KETOPROFEN | Tablet/Suppository/ Capsule |
| KETOROLAC TROMETHAMINE | Liquid-IM / Tablet |
| MEFENAMIC ACID | Capsule |
| MELOXICAM | Tablet |
| NABUMETONE | Tablet |
| NAPROXEN | Tablet / Suppository |
| OXAPROZIN | Tablet |
| OXYPHENBUTAZONE | Tablet |
| PHENYLBUTAZONE | Tablet |
| PIROXICAM | Capsule / Suppository |
| ROFECOXIB | Tablet |
| SULINDAC | Tablet |
| TENOXICAM | Tablet |
| TIAPROFENIC ACID | Tablet / Capsule |
| TOLMETIN | Tablet |
| TOLMETIN SODIUM | Capsule |
| VALDECOXIB | Tablet |
| 1. ***Opioids*** | |
| ACETAMINOPHEN /CHLORPHENIRAMINE MALEATE/CODEINE PHOSPHATE/ PSEUDOEPHEDRINE HYDROCHLORIDE | Tablet |
| ACETAMINOPHEN/ CODEINE PHOSPHATE | Tablet/ Elixir |
| ACETAMINOPHEN/CAFFEINE CITRATE/ CODEINE PHOSPHATE | Tablet |
| ACETAMINOPHEN/CAFFEINE/CODEINE PHOSPHATE/DIPHENYLPYRALINE HYDROCHLORIDE/ PHENYLPROPANOLAMINE HYDROCHLORIDE | Tablet |
| ACETAMINOPHEN/CHLORZOXAZONE/ CODEINE PHOSPHATE | Tablet |
| ACETAMINOPHEN/CODEINE PHOSPHATE/ METHOCARBAMOL | Tablet |
| ACETAMINOPHEN/CODEINE PHOSPHATE/DOXYLAMINE SUCCINATE | Tablet |
| ACETAMINOPHEN/OXYCODONE HYDROCHLORIDE | Tablet |
| ACETAMINOPHEN/TRAMADOL HCl | Tablet |
| ACETYLSALICYLIC ACID /CAFFEINE /CODEINE PHOSPHATE | Tablet |
| ACETYLSALICYLIC ACID /CAFFEINE /DEXTROPROPOXYPHENE HCL(NAPSYLATE) | Tablet |
| ACETYLSALICYLIC ACID /CODEINE PHOSPHATE | Tablet |
| ACETYLSALICYLIC ACID /CODEINE PHOSPHATE/METHOCARBAMOL | Tablet |
| ACETYLSALICYLIC ACID/ BUTALBITAL/ CAFFEINE/ CODEINE PHOSPHATE | Capsule |
| ACETYLSALICYLIC ACID/ALUMINUM HYDROXIDE/CAFFEINE/CODEINE PHOSPHATE/ MAGNESIUM HYDROX | Tablet |
| ACETYLSALICYLIC ACID/CAFFEINE CITRATE/ CODEINE PHOSPHATE/ MEPROBAMATE | Tablet |
| ACETYLSALICYLIC ACID/CODEINE PHOSPHATE/ PHENOBARBITAL | Capsule |
| ACETYLSALICYLIC ACID/OXYCODONE HYDROCHLORIDE | Tablet |
| ALCOHOL ANHYDROUS/AMMONIUM CHLORIDE /CODEINE PHOSPHATE /GUAIFENESIN | Syrup |
| ALFENTANIL (ALFENTANIL HYDROCHLORIDE) | Solution |
| AMMONIUM ACETATE /CODEINE PHOSPHATE /GUMWEED /MENTHOL /SQUILL | Syrup |
| AMMONIUM CHLORIDE/BROMODIPHENHYDRAMINE HYDROCHLORIDE /CODEINE PHOSPHATE /DIPHENHYDRAMINE HYDROCHLORIDE /POTASSIUM GUAIACOL SULPHONATE | Liquid |
| AMMONIUM CHLORIDE/CODEINE PHOSPHATE/DIPHENHYDRAMINE HYDROCHLORIDE | Syrup/Liquid |
| ANILERIDINE (ANILERIDINE PHOSPHATE) | Liquid/Tablet |
| ATROPINE SULFATE /ATTAPULGITE (ACTIVATED) /HYOSCYAMINE SULFATE /OPIUM/PECTIN /SCOPOLAMINE HYDROBROMIDE | Capsule |
| ATROPINE SULFATE/ATTAPULGITE (ACTIVATED) /HYOSCYAMINE SULFATE /OPIUM /PECTIN /SCOPOLAMINE HYDROBROMIDE | Capsule |
| ATTAPULGITE (ACTIVATED) /OPIUM /PECTIN | Capsule |
| BELLADONNA /OPIUM | Suppository |
| BENZOIC ACID/CAMPHOR/OPIUM | Tincture |
| BROMPHENIRAMINE MALEATE /CODEINE PHOSPHATE/ PHENYLEPHRINE HYDROCHLORIDE /PHENYLPROPANOLAMINE HYDROCHLORIDE | Syrup |
| BROMPHENIRAMINE MALEATE/CODEINE PHOSPHATE /GUAIFENESIN /PHENYLEPHRINE HYDROCHLORIDE /PHENYLPROPANOLAMINE HYDROCHLORIDE | Syrup |
| BUPRENORPHINE (BUPRENORPHINE HCL) | Patch/Film,Soluble |
| BUPRENORPHINE HCL/NALOXONE | Tablet/Film,Soluble |
| BUPRENORPHINE HYDROCHLORIDE | Tablet/Implant/Solution (extended release) |
| BUTORPHANOL TARTRATE | Solution/Spray/Aerosol-metered dose, Liquid |
| CAMPHOR/OPIUM | Tincture |
| CAMPHOR/OPIUM/TANNIC ACID | Tablet |
| CHLORPHENIRAMINE MALEATE/CODEINE | Syrup |
| CHLORPHENIRAMINE/CODEINE /EPHEDRINE AS RESIN COMPLEX/GUAIACOL CARBONATE/ PHENYLTOLOXAMINE | Syrup |
| COCILLANA/CODEINE PHOSPHATE /EUPHORBIA/SENEGA/SQUILL/WILD LETTUCE | Syrup |
| CODEINE MONOHYDRATE, CODEINE SULFATE TRIHYDRATE | Tablet |
| CODEINE PHOSPHATE | Syrup/ Tablet/Solution-Liquid |
| CODEINE PHOSPHATE /PHENYLEPHRINE HYDROCHLORIDE | Syrup |
| CODEINE PHOSPHATE/ GUAIFENESIN/ PSEUDOEPHEDRINE HYDROCHLORIDE | Syrup |
| CODEINE PHOSPHATE/ GUAIFENESIN/ PSEUDOEPHEDRINE HYDROCHLORIDE/ TRIPROLIDINE HYDROCHLORIDE | Syrup |
| CODEINE PHOSPHATE/ PHENYLEPHRINE HYDROCHLORIDE/ POTASSIUM GUAIACOL SULPHONATE /PROMETHAZINE HYDROCHLORIDE | Liquid |
| CODEINE PHOSPHATE/ PSEUDOEPHEDRINE HYDROCHLORIDE/ TRIPROLIDINE HYDROCHLORIDE | Syrup |
| CODEINE PHOSPHATE/DIPHENYLPYRALINE HYDROCHLORIDE/PHENYLEPHRINE HYDROCHLORIDE | Syrup |
| CODEINE PHOSPHATE/GUAIFENESIN/PHENIRAMINE MALEATE | Syrup |
| CODEINE PHOSPHATE/IPECAC /POTASSIUM CITRATE /SQUILL | Syrup |
| CODEINE PHOSPHATE/PHENIRAMINE MALEATE /PHENYLPROPANOLAMINE HYDROCHLORIDE /PYRILAMINE MALEATE | Syrup |
| CODEINE PHOSPHATE/PHENIRAMINE MALEATE/PHENYLPROPANOLAMINE HYDROCHLORIDE/PYRILAMINE MALEATE | Syrup |
| CODEINE PHOSPHATE/POTASSIUM GUAIACOL SULPHONATE /PROMETHAZINE HYDROCHLORIDE | Liquid |
| CODEINE PHOSPHATE/PSEUDOEPHEDRINE HYDROCHLORIDE /TRIPROLIDINE HYDROCHLORIDE | Liquid |
| CODEINE PHOSPHATE/PSEUDOEPHEDRINE HYDROCHLORIDE/TRIPROLIDINE HYDROCHLORIDE | Syrup/Tablet |
| CODEINE/TERPIN HYDRATE | Syrup |
| DEXTROPROPOXYPHENE HYDROCHLORIDE | Tablet/Capsule |
| DIAMORPHINE | Powder for solution |
| DROPERIDOL /FENTANYL CITRATE | Liquid |
| FENTANYL | Patch |
| FENTANYL (FENTANYL CITRATE) | Film,Soluble/Tablet/Solution |
| FENTANYL CITRATE | Liquid/Solution |
| HYDROMORPHONE HYDROCHLORIDE | Tablet/ Suppository/powder for solution/Solution-Liquid/Syrup/Capsule |
| KAOLIN/OPIUM /PECTIN | Liquid |
| MEPERIDINE HCL | Solution/Liquid |
| MORPHINE HYDROCHLORIDE | Syrup/Suppository/Tablet |
| MORPHINE SULFATE | Liquid/Solution/ Suppository/ Tablet/Capsule/Drops/Syrup |
| NALBUPHINE HYDROCHLORIDE | Solution |
| NALOXONE HYDROCHLORIDE/OXYCODONE HYDROCHLORIDE | Tablet |
| OPIUM | Tincture |
| OPIUM (MORPHINE) | Tincture |
| OXYCODONE HYDROCHLORIDE | Suppository/Tablet |
| OXYMORPHONE HYDROCHLORIDE | Liquid/Suppository/Tablet |
| PENTAZOCINE (PENTAZOCINE HYDROCHLORIDE) | Tablet |
| PENTAZOCINE (PENTAZOCINE Lactate) | Liquid/Solution |
| REMIFENTANIL (REMIFENTANIL HCL) | Powder for Solution |
| SUFENTANIL (SUFENTANIL CITRATE) | Liquid |
| TAPENTADOL (TAPENTADOL HYDROCHLORIDE) | Tablet |
| TRAMADOL HCL | Tablet/Capsule |
| 1. ***Respiratory medications*** | |
| ACLIDINIUM BROMIDE | Powder |
| AMINOPHYLLINE | Tablet/Solution |
| BECLOMETHASONE DIPROPIONATE | Capsule/Powder/metered dose |
| BENRALIZUMAB | Solution |
| BUDESONIDE | Suspension/Powder |
| CICLESONIDE | METERED DOSE |
| CROMOLYN SODIUM | Solution/Liquid/metered dose |
| DEXTROSE/THEOPHYLLINE | Solution |
| DYPHYLLINE | Elixir/Solution/Tablet |
| EPHEDRA | Capsule |
| EPHEDRINE HCL | Tablet/Capsule/Solution |
| EPINEPHRINE | Metered dose/Spray/Solution |
| FENOTEROL HYDROBROMIDE | Metered dose/Tablet/Solution |
| FLUNISOLIDE | Metered dose |
| FLUTICASONE FUROATE | Powder/Suspension/metered dose/Drops |
| FLUTICASONE PROPIONATE/SALMETEROL | Powder/metered dose |
| FORMOTEROL FUMARATE | Capsule |
| FORMOTEROL FUMARATE/MOMETASONE | Metered dose |
| FORMOTEROL FUMARATE/GLYCOPYRRONIUM | Metered dose |
| GLYCOPYRRONIUM | Powder |
| GUAIFENESIN/OXTRIPHYLLIN | Elixir |
| GUAIFENESIN/POTASSIUM IODIDE/PYRILAMINE /THEOPHYLLINE | Syrup/Tablet |
| IBUPROFEN/PSEUDOEPHEDRINE HCL | Tablet |
| INDACATEROL /MOMETASONE FUROATE | Capsule |
| INDACATEROL | Capsule |
| IPRATROPIUM BROMIDE | Liquid/Solution |
| IPRATROPIUM BROMIDE/SALBUTAMOL | Solution/Liquid/metered dose |
| ISOPROTERENOL | Liquid/metered dose |
| L-EPHEDRINE | Tablet/Capsule |
| MEPOLIZUMAB | Solution/Powder |
| MOMETASONE FUROATE | Powder/Spray |
| MONTELUKAST | Tablet |
| NEDOCROMIL SODIUM | Metered dose |
| OLODATEROL | Solution |
| OMALIZUMAB | Solution/Powder |
| ORCIPRENALINE | Metered dose/Syrup/Liquid/Tablet |
| OXTRIPHYLLINE | Syrup/Elixir/Tablet |
| PIRBUTEROL | Metered dose |
| PROCATEROL | Metered dose |
| SALBUTAMOL | Solution/Liquid/metered dose/Tablet |
| SALMETEROL | Metered dose/Powder |
| TERBUTALINE | Powder/Tablet |
| Theophylline | Tablet/Capsule/Elixir/Solution |
| TIOTROPIUM | Capsule/Solution |
| TRIAMCINOLONE ACETONIDE | Liquid/Metered dose |
| UMECLIDINIUM | Powder |
| ZAFIRLUKAST | Tablet |
| 1. ***Chemotherapy Medications*** | |
| ABEMACICLIB | Tablet |
| ACALABRUTINIB | Capsule |
| AFATINIB | Tablet |
| AFLIBERCEPT | Solution |
| ALECTINIB | Capsule |
| ALPELISIB | Tablet |
| ALTRETAMINE | Capsule |
| AMINOLEVULINIC ACID HCL | Powder |
| AMSACRINE | Liquid |
| ANAGRELIDE | Capsule |
| ARSENIC TRIOXIDE | Solution |
| ASPARAGINASE | Powder |
| ATEZOLIZUMAB | Solution |
| AVELUMAB | Solution |
| AXICABTAGENE CILOLEUCEL | Suspension |
| AXITINIB | Tablet |
| AZACITIDINE | Powder |
| BENDAMUSTINE HCL | Powder |
| BEVACIZUMAB | Solution |
| BLEOMYCIN | Powder |
| BLINATUMOMAB | Powder |
| BORTEZOMIB | Powder |
| BOSUTINIB | Tablet |
| BRENTUXIMAB VEDOTIN | Powder |
| BRIGATINIB | Tablet |
| BUSULFAN | Tablet/Solution |
| CABAZITAXEL | Solution |
| CABOZANTINIB | Tablet |
| CAPECITABINE | Tablet |
| CARBOPLATIN | Liquid/Solution/Powder |
| CARFILZOMIB | Powder |
| CARMUSTINE | Powder |
| CATUMAXOMAB | Solution |
| CEDAZURIDINE/DECITABINE | Tablet |
| CEMIPLIMAB | Solution |
| CERITINIB | Capsule |
| CETUXIMAB | Solution |
| CHLORAMBUCIL | Tablet |
| CISPLATIN | Liquid/Solution |
| CLADRIBINE | Solution |
| CLOFARABINE | Solution |
| COBIMETINIB | Tablet |
| CRIZOTINIB | Capsule |
| CYCLOPHOSPHAMIDE | Powder/Tablet |
| CYTARABINE | Powder/Solution/Suspension |
| DABRAFENIB | Capsule |
| DACARBAZINE | Powder/Liquid |
| DACOMITINIB | Tablet |
| DACTINOMYCIN | Powder |
| DARATUMUMAB | Solution |
| DASATINIB | Tablet |
| DAUNORUBICIN | Suspension/Solution/Powder |
| DECITABINE | Powder |
| DINUTUXIMAB | Solution |
| DOCETAXEL | Solution |
| DOXORUBICIN HCL | Powder/Solution/Suspension/Liposomes |
| DURVALUMAB | Solution |
| ELOTUZUMAB | Powder |
| ENASIDENIB | Tablet |
| ENTRECTINIB | Capsule |
| EPIRUBICIN HCL | Powder/Solution |
| ERDAFITINIB | Tablet |
| ERIBULIN MESYLATE | Solution |
| ERLOTINIB | Tablet |
| ESTRAMUSTINE PHOSPHATE SODIUM | Capsule |
| ETOPOSIDE | Liquid/Capsule/Solution |
| EVEROLIMUS | Tablet/ |
| FEDRATINIB | Capsule |
| FLUDARABINE PHOSPHATE | Tablet/Solution/Powder |
| FLUOROURACIL | Solution/Cream/Powder |
| GEFITINIB | Tablet |
| GEMCITABINE | Powder |
| GEMTUZUMAB OZOGAMICIN | Powder |
| GILTERITINIB | Tablet |
| GLASDEGIB | Tablet |
| HYDROXYUREA | Capsule |
| IBRUTINIB | Capsule/Tablet |
| IDARUBICIN HCL | Powder/Solution/Capsule/Tablet |
| IDELALISIB | Tablet |
| IFOSFAMIDE | Powder |
| IMATINIB | Capsule/Tablet |
| INOTUZUMAB OZOGAMICIN | Powder |
| IPILIMUMAB | Liquid |
| IRINOTECAN | Suspension/Solution |
| ISATUXIMAB | Solution |
| IXAZOMIB | Capsule |
| LAPATINIB | Tablet/Capsule/Solution |
| LAROTRECTINIB | Capsule/Solution |
| LENVATINIB | Capsule |
| LEUCOVORIN CALCIUM/TEGAFUR /URACIL | Capsule |
| LOMUSTINE | Capsule |
| LORLATINIB | Tablet |
| MELPHALAN | Tablet |
| MERCAPTOPURINE | Tablet |
| METHOTREXATE | Tablet/Liquid/Solution |
| MIDOSTAURIN | Capsule |
| MITOMYCIN | Powder |
| MITOTANE | Tablet |
| MITOXANTRONE | Liquid/Solution |
| NECITUMUMAB | Solution |
| NELARABINE | Solution |
| NERATINIB | Tablet |
| NILOTINIB | Capsule |
| NINTEDANIB | Capsule |
| NIRAPARIB | Capsule |
| NIVOLUMAB | Solution |
| OBINUTUZUMAB | Solution |
| OFATUMUMAB | Solution |
| OLAPARIB | Tablet/Capsule |
| OLARATUMAB | Solution |
| OSIMERTINIB | Tablet |
| OXALIPLATIN | Powder/Solution |
| PACLITAXEL | Powder/Solution/Liquid |
| PALBOCICLIB | Capsule/Tablet |
| PANITUMUMAB | Solution |
| PAZOPANIB | Tablet |
| PEGASPARGASE | Liquid/Solution |
| PEMBROLIZUMAB | Solution/Powder |
| PEMETREXED | Powder |
| PENTOSTATIN | Powder |
| PERTUZUMAB | Solution |
| POLATUZUMAB VEDOTIN | Solution |
| PONATINIB | Tablet |
| PORFIMER SODIUM | Powder |
| PRALATREXATE | Solution |
| PROCARBAZINE | Capsule |
| RALTITREXED | Powder |
| RAMUCIRUMAB | Solution |
| REGORAFENIB | Tablet |
| RIBOCICLIB | Tablet |
| RITUXIMAB | Solution |
| ROMIDEPSIN | Powder |
| RUXOLITINIB | Tablet |
| SONIDEGIB | Capsule |
| SORAFENIB | Tablet |
| STREPTOZOCIN | Powder |
| SUNITINIB | Capsule |
| TALAZOPARIB | Capsule |
| TAMOXIFEN | Tablet |
| TEMOZOLOMIDE | Powder/Capsule |
| TEMSIROLIMUS | Liquid/Solution |
| TENIPOSIDE | Liquid |
| THIOGUANINE | Tablet |
| THIOTEPA | Powder |
| TIPIRACIL /TRIFLURIDINE | Tablet |
| TOPOTECAN | Powder/Solution |
| TRABECTEDIN | Powder |
| TRAMETINIB | Tablet |
| TRASTUZUMAB | Powder/Solution |
| TRETINOIN | Capsule |
| TUCATINIB | Tablet |
| VALRUBICIN | Solution |
| VEMURAFENIB | Tablet |
| VENETOCLAX | Tablet |
| VINBLASTINE SULFATE | Liquid/Solution/Powder |
| VINCRISTINE | Liquid/Solution/Powder |
| VINDESINE SULFATE | Powder |
| VINORELBINE | Solution |
| VISMODEGIB | Capsule |
| VORINOSTAT | Capsule |
| 1. ***Proton Pump Inhibitors*** | |
| DEXLANSOPRAZOLE | Capsule |
| ESOMEPRAZOLE | Tablet/capsule |
| ESOMEPRAZOLE/NAPROXEN | Tablet |
| LANSOPRAZOLE | Tablet/capsule/Granule/powder |
| OMEPRAZOLE | Tablet |
| PANTOPRAZOLE | Tablet/powder |
| RABEPRAZOLE | Tablet |
| AMOXICILLIN/CLARITHROMYCIN/LANSOPRAZOLE | Capsule |
| 1. ***Thyroid*** | |
| LEVOTHYROXINE | Tablet/Liquid/powder |
| LIOTHYRONINE | Tablet |
| PARATHYROID HORMONE | Drops |
| THYROID | Tablet/Liquid/Drops |
| ADRENAL CORTICAL EXTRACT/ORCHITIC EXTRACT/PANCREATIC EXTRACT/THYROID/WILD THYME | Liquid |
| CALCIUM PHOSPHATE/FUCUS VESICULOSUS/LUPULUS/POTASSIUM IODIDE/THYROID | Tablet |
| 1. ***Statins*** | |
| ATORVASTATIN | Tablet |
| AMLODIPINE/ATORVASTATIN | Tablet |
| CERIVASTATIN | Tablet |
| FLUVASTATIN | Tablet/capsule |
| LOVASTATIN | Tablet |
| LOVASTATIN/NICOTINIC ACID | Tablet |
| PRAVASTATIN | Tablet |
| ACETYLSALICYLIC ACID/PRAVASTATIN | Tablet |
| ROSUVASTATIN | Tablet |
| SIMVASTATIN | Tablet |
| 1. ***Alpha-1 adrenergic receptor blockers*** | |
| ALFUZOSIN | Tablet |
| DOXAZOSIN | tablet |
| DUTASTERIDE/TAMSULOSIN | Capsule |
| PRAZOSIN | Tablet |
| SILODOSIN | Capsule |
| TAMSULOSIN HYDROCHLORIDE | Tablet/capsule |
| TERAZOSIN | Tablet |
| 1. ***Bisphosphonates*** | |
| ALENDRONIC ACID | Tablet/solution |
| ALENDRONIC ACID/VITAMIN D3 | Tablet |
| CLODRONATE DISODIUM | Capsule/solution |
| ETIDRONATE DISODIUM | Tablet/solution |
| IBANDRONIC ACID | Liquid |
| PAMIDRONATE DISODIUM | Liquid/solution/powder |
| RISEDRONATE SODIUM | Tablet |
| ZOLEDRONIC ACID | Solution/powder |

**ICD codes for comorbid diseases:**

1. **Coronary artery dieses:** ICD9 codes (410-414), ICD10 codes (I20-I25).
2. **Asthma:** ICD-9-CM codes (493.00 – 493.99), ICD-10-CM codes (J45.0 – J45.998)
3. **Congestive Heart Failure:**  ICD-9-CM (428), ICD-10-CA (150.0, I50.1, I50.9, I13.0, I13.2).
4. **Hypertension:** ICD-9-CM (401-405), ICD-10-CA (I10-I13, I15).
5. **Diabetes:** ICD-9-CM (250), ICD-10-CA (E10-E14).
